# Anti-Emetics in Children Receiving Chemotherapy for Solid Tumors and Leukemia: Pharmacology and Optimization of Therapy for Nausea and Vomiting

**DOI:** 10.3390/ph17050616

**Published:** 2024-05-10

**Authors:** Shuvadeep Ganguly, Archana Sasi, Santhosh Kumar Kodagalli Nagaraju, Sameer Bakhshi

**Affiliations:** Department of Medical Oncology, Dr. B.R.A. Institute Rotary Cancer Hospital, All India Institute of Medical Sciences, New Delhi 110029, India; ganguly.shuvadeep@gmail.com (S.G.); archana.311094@gmail.com (A.S.); santhoshonco123@gmail.com (S.K.K.N.)

**Keywords:** children, anti-emetic, olanzapine, dexamethasone, aprepitant, drug interaction, chemotherapy

## Abstract

The management of chemotherapy-induced nausea and vomiting (CINV) in children remains challenging due to differences in the chemotherapy regimens, their relative emetogenicity compared to that in adults and differences in drug metabolism and the available formulations. The common four classes of anti-emetics used for the treatment and prophylaxis of CINV in children include dexamethasone, neurokinin-1 receptor antagonists, 5-hydroxytryptamine-3 receptor antagonists (5HT3RAs), and olanzapine. The appropriate dose of dexamethasone for CINV prophylaxis in children is unknown, with a significant variability in dosage ranging between 6 and 32 mg/m^2^/day. The dose of dexamethasone is decreased by 30% when this drug is combined with (fos)aprepitant in children, in contrast to a decrease of 50% required in adults. The use of aprepitant in younger children (<12 years) is often hampered by the non-availability of oral suspension formulations in many countries; alternatively, 80 mg capsules are administered for 1–3 days in certain institutes to children weighing between 15 and 40 kg. Among the different 5HT3RAs, palonosetron is comparatively metabolized faster in children than in adults, requiring a higher dosage for similar efficacy to that achieved in adults. Olanzapine is a newer agent, used in doses between 0.1 and 0.14 mg/kg/day in children, with good anti-emetic efficacy, but has sedation and hyperglycemia as concerning adverse effects. Drug interactions between anti-emetics and between anti-emetics and chemotherapy/supportive agents (azole antifungals, cyclosporine, arsenic trioxide), especially QTc prolongation, should be considered during prescription.

## 1. Introduction

Chemotherapy-induced nausea and vomiting (CINV) is a common debilitating adverse effect affecting up to 70% of children receiving anti-neoplastic therapy [1]. Chemotherapy-induced vomiting (CIV) has a multifactorial etiology mediated by multiple neurotransmitters. The acute phase of CIV (from the initiation of chemotherapy to 24 h after the completion of chemotherapy) is predominantly mediated by 5-hydroxytryptamine (5-HT3), while the delayed phase of CIV (between 24 h and 120 h after the completion of chemotherapy) is mediated by substance P and dopamine, although this distinction is not absolute [2].

The true incidence of delayed and anticipatory CINV in children remains unclear, due to difficulties in the assessment of nausea in children [3,4,5]. The extrapolation of the efficacy of anti-emetics in adults to predict that in children is also erroneous due to the differing metabolism of certain drugs in children and adults. The chemotherapy protocols in children are often intensive and multi-day, unlike adult protocols, which are largely single-day. Yet, pediatric guidelines and dosing strategies for anti-emetic prophylaxis are largely derived by extrapolation from studies conducted in adults [6]. There is a need for clinicians to better understand the pharmacology of anti-emetics in children to facilitate CINV control by optimizing the dosing strategies in this population. In this review, the authors attempt to elucidate the available evidence on the pharmacology of anti-emetics in the pediatric population in the context of the prophylaxis and management of CINV.

## 2. Methodology

A literature search was conducted in PubMed using the keywords “nausea” OR “vomiting” OR “chemotherapy-induced nausea and vomiting” OR “anti-emetics” OR “5-HT3 antagonists” OR “Dexamethasone” OR “Olanzapine” AND “polymorphism” OR “pharmacokinetics” OR “drug interactions” AND “children” OR “Pediatric”. A total of 1756 studies were retrieved. The authors independently assessed the relevance of the studies to the review objective based on the title/abstract, followed by full text screening, and relevant studies were selected for this narrative review. There was no restriction on the type of study or the year of publication. Additionally, the guidelines for the management of CINV, especially focusing on children, were purposively reviewed. The data and conclusions of the included studies were synthesized topic-wise for this narrative review.

## 3. Risk Factors for CINV among Children

Among children, older age and susceptibility to motion sickness have been consistently identified as risk factors for acute CINV [7,8,9]. Unlike what is seen in adults, no gender-based differences in the perception of CINV were identified [8,10]. Disease-specific analyses showed that children with hematological malignancies are more likely to experience delayed CINV compared to those with solid tumors, likely due to the variable use of steroids in this population in the actual treatment [8]. Multi-day chemotherapy protocols commonly used in children lead to longer acute-phase duration, which has been associated with poorer acute-phase CIV control, likely due to the overlap of different phases [3]. 

A genetic association study conducted in children showed that genetic polymorphisms like those in the 5HT-3 receptor gene (*HT3RB*) and dopamine transport gene *SLC6A3* were associated with poor response to anti-emetic prophylaxis for CINV [11]. Similarly, rapid-metabolizer polymorphisms in *CYP2D6*, the metabolizing enzyme for 5-HT3 antagonists, are shown to be associated with reduced ondansetron efficacy against post-operative nausea and vomiting among adults, while the effect of *CYP2D6* rapid-metabolizer status did not show a statistical significant relation with ondansetron efficacy in children [12,13]. However, the clinical implications of such polymorphisms for dosing strategies, especially for children, against CINV, still remain unclear.

## 4. Pharmacology and Kinetics of the Anti-Emetics in Use in Children

**Figure 1:**
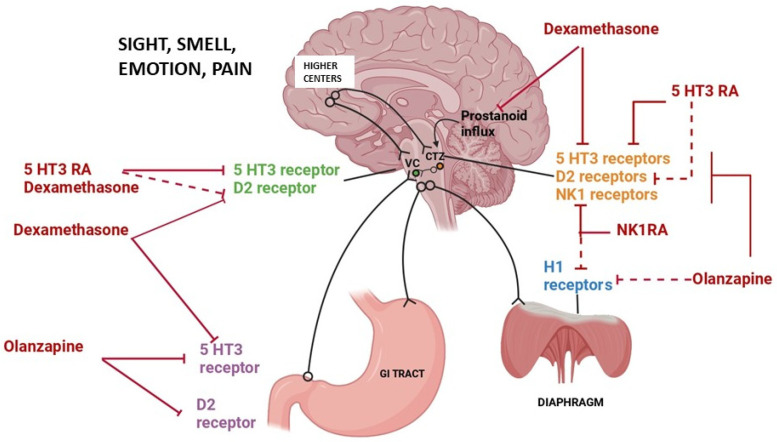
Mechanism of chemotherapy-induced nausea and vomiting and site of action of different classes of anti-emetics. Solid lines: predominant mechanisms of action; dashed lines: minor pathways of action.

The following class of anti-emetic agents are currently in use for children for the prophylaxis and management of CINV: dexamethasone, 5-HT3 antagonists, neurokinin-antagonists, and olanzapine. The mechanisms of action of various classes of anti-emetics are shown in Figure 1. The available formulations and dosing strategies for children are shown in Table 1. Additionally, clinically significant incompatibilities in combining intravenous formulations and/or contraindications of the use of specific agents are mentioned [14,15,16]. With the available evidence of efficacy of newer agents, the use of older anti-emetic agents like prochlorperazine and domperidone is currently comparatively sparse in children for the management of CINV. The current status of the use of other agents like benzodiazepines, medical cannabinoids or traditional medicines as anti-emetics in children for CINV is also discussed briefly.

### 4.1. Dexamethasone

Among steroids, both dexamethasone and methylprednisolone are known to have similar anti-emetic efficacy; yet, the use of methylprednisolone as a routine anti-emetic has declined because of the better CNS penetration and longer action of dexamethasone.

*Pharmacology:* The precise anti-emetic action of dexamethasone is unclear. It acts as an antagonist via 5HT3 receptors, its action being noncompetitive with and additive to that of serotonin antagonists. Dexamethasone also inhibits prostanoid synthesis and influx into chemoreceptor zones in the medulla oblongata. Steroids may act as anti-emetics by decreasing the acute hypothalamo–pituitary axis suppression induced by chemotherapeutic agents [38].

Dexamethasone has oral bioavailability of 86% and should have similar efficacy when administered by the oral and intravenous routes. The elimination half-life of dexamethasone is higher in children or infants (3–8 h and 2–10 h, respectively) compared to adults (3 h) [39].

*Pediatric dosing and formulations*: The optimal dosing of dexamethasone for CINV prophylaxis is not known. In a systematic review, the dosing regimens varied from 6 to 27 mg/m^2^/day in patients receiving highly emetogenic chemotherapy (HEC) and from 0.6 to 24 mg/m^2^/day in patients receiving moderately emetogenic chemotherapy (MEC). While the optimal dosing could not be recommended due to the large heterogeneity of the studies, a dose of 6 mg/m^2^/day appears efficacious [17]. In adults, in combination with aprepitant, the clearance of dexamethasone is decreased by 50% with 125 mg of aprepitant and by 25% with 40 mg of aprepitant [40]. In children, the clearance is not affected similarly, and hence, when administered with aprepitant, the IV dexamethasone dosage should be reduced by 30% [18].

For HEC and MEC, dexamethasone is continued for 3 days beside the pre-chemotherapy dose when combined with 5HT3 antagonists with/without neurokinin antagonists. For adults receiving 20 mg of dexamethasone on day 1, combined with 0.25 mg of palonosetron on day 1, it was observed that steroids can be omitted from day 2 onwards [41]. However, the possibility of dexamethasone omission while receiving palonosetron has not been shown in children.

*Adverse effects*: During intravenous injection, dexamethasone causes dose-dependent perineal pruritis, mitigated by dilution in 50 mL of saline and a slow push over 15–30 min. A specific study among adults to assess the adverse effects of dexamethasone when used in CINV prophylaxis showed that one in two persons experienced insomnia, around 27% of the patients experienced epigastric discomfort, agitation and insomnia, and around 15–20% experienced increased appetite, weight gain and acne [41]. A study conducted among children that assessed the safety of dexamethasone among those undergoing hematopoietic stem cell transplantation, adverse effects like hyperglycemia, hypertension, bradycardia, dyspepsia, gastroesophageal reflux and mood alterations were seen, although none of the adverse effects were above grade III [42].

In adults, the use of a dexamethasone-free approach consisting in the administration of a netupitant–palonosteron combination or in the substitution of dexamethasone with olanzapine was shown to have similar efficacy as the use of dexamethasone; however, prospective data are awaited for children [43].

### 4.2. HT3 Receptor Antagonists

The 5HT3 receptor antagonists are the drugs most commonly used for the prevention and management of CINV for both children and adults. The approved agents for children include first-generation ondansetron, granisetron, tropisetron, dolasetron and second-generation palonosetron. The approval of dolasetron against CINV was withdrawn by the United States Food and Drug Administration due to concerns regarding QTc prolongation [44].

*Pharmacology:* The 5HT3 receptors are centrally responsible for CINV and are the targets of multiple classes of anti-emetics including 5HT3 receptor antagonists (5HT3RAs), dopamine antagonists; they also mediate the off-target action of dexamethasone and olanzapine (Figure 1).

The different 5HT3RAs show broadly similar efficacy; yet, they differ significantly pharmacologically. These agents differ maximally in their oral bioavailability, elimination half-life and receptor affinity. The half-life is the shortest for ondansetron (3.5–5.5 h), followed by the other first-generation agents granisetron (4.5 h to 7.5 h), dolasetron (7.3 h), tropisetron (5.5 h). The oral bioavailability of ondansetron is 56%, with the bioavailability of other first-generation agents ranging between 65% and 75%. In contrast, the half-life of palonosetron is 40 h in adults, with bioavailability of 10% due to extensive first-pass metabolism precluding its oral administration [37,45]. However, for palonosetron, the clearance half-life is significantly higher in adults compared to children (half-life of 29 h in children), leading to higher dosing being necessary to achieve a similar systemic exposure [36].

*Pediatric dosing and formulations*: The available formulations and dosing strategies of all the available agents are reported in Table 1. The dosing strategies have been variable, often limited by the availability of the formulations [33,34,35]. The subcutaneous extended-release formulation and transdermal patch of granisteron which are approved for adults are currently not approved for children. For orally administered ondansetron and granisetron, the time to the peak blood concentration after oral ingestion is between 0.5 and 2 h; hence, oral administration should be performed at least 30 min–1 h prior to chemotherapy [32]. Compared to ondansetron, other first-generation 5HT3RAs have a longer half-life and, hence, are used in a single daily dose, while ondansetron is commonly administered two–three times per day. However, receptor blockade does not correlate with the elimination half-life and all 5-HT3 antagonists can be administered once daily, with similar efficacy [46]. However, for highly emetogenic chemotherapy, a multiple daily dosing of ondansetron may be more beneficial [31].

*Adverse effects:* Overall, 5HT3RAs are well tolerated, although concerns for cardiac safety due to QTc prolongation and drug–drug interactions remain. Headache, constipation and asthenia are common adverse effects recorded in the literature, although their clinical significance is minimal [45]. In a systematic review and network meta-analysis which comparatively assessed the safety of different 5HT3RAs in patients receiving chemotherapy, these agents were found to be relatively safe and well tolerated, with no increase in arrythmia or mortality; however, in a network meta-analysis, the risk of arrythmia was higher in patients receiving dolasetron in comparison to those administered ondansetron; the adverse effects of 5HT3RAs were not found to differ between children and adults [47].

### 4.3. Neurokinin-1 Receptor Antagonists

NK-1 receptor antagonists include agents like aprepitant, fosaprepitant, rolapitant, netupitant, which form a new class of anti-emetics for the management of CINV. Of the above agents, aprepitant and fosaprepitant are approved for use in children [21,22].

*Pharmacology of aprepitant:* Human substance P is a nociceptive neurotransmitter mediating the action of CINV through NK-1 receptors centrally present in the nucleus of tractus solitarius and area postrema and peripherally present in the gastrointestinal tract [48]. Aprepitant is a highly selective antagonist of the NK-1 receptor that is effective for both acute and possibly delayed CINV.

The oral absorption of aprepitant peaks at 4 h and shows non-linear kinetics, with decreasing absorption at higher doses [49]. Hence, the oral dose should be administered at least 1–2 h before chemotherapy. It is metabolized by CYP3A4 and CYP2C9 and is a competitive inhibitor of CYP3A4, with significant potential for drug–drug interactions.

*Pediatric dosing and formulations*: Aprepitant is available as an oral capsule formulation of 125 mg and 80 mg, which can be used for children who are able to swallow capsules. For children unable to swallow capsule, an oral suspension of aprepitant (25 mg/mL) is available in certain countries for children above 6 months for the administration of a dose of 3 mg/kg on day 1 and 2 mg/kg on day 2 and day 3 [21,50]. Aprepitant is poorly water-soluble and has an oral bioavailability of 59%; hence, the direct dissolution of the capsule formulation in water is discouraged [51]. An alternate dosing strategy was used at the authors’ institution, where children (5–18 years) between 15 and 40 kg received 80 mg capsules from day 1 to day 3, instead of the adult dose, which was safe and well tolerated [22]. However, for smaller children unable to swallow capsules, the non-availability of an oral suspension remains a concern in many countries. An extemporaneously prepared aprepitant oral suspension formulation showed an oral bioavailability of 82.3% relative to that of capsules in 17 healthy volunteers, and its use may be explored among children or adults unable to swallow capsules [50].

*Pharmacology of fosaprepitant*: Fosaprepitant is a phosphoryl prodrug of aprepitant that is water-soluble and rapidly converted within 30 min to aprepitant within the body; a dose of 115 mg of fosaprepitant in oral capsule is equivalent to a dose of 125 mg of aprepitant [25].

*Pediatric dosing and formulations*: For fosaprepitant, a pharmacokinetic study conducted among 2–17-year-old children showed that for the age group older than 12 years, a dose of 150 mg provided a similar adult exposure. For children of less than 12 years, a higher dose (up to 5 mg/kg) was necessary to achieve a similar adult exposure, due to enhanced clearance in children [26]. The approved dose of fosparepitant is 4 mg/kg for patients between 2 and 12 years of age and 5 mg/kg for patients between 6 months and 2 years of age. Clinical trials used single intravenous doses between 3 and 4 mg/kg on day 1 [24,52]. The relative lower efficacy of fosaprepitant in children compared to adults may be related to a lower systemic exposure at a similar relative dose. Hence, the use of fosaprepitant for 3 days for multi-day chemotherapy and of oral aprepitant formulations on day 2 and day 3 after an intravenous dose of fosaprepitant on day 1 is recommended by the manufacturer and was being explored in studies, with unclear efficacy [53]. While intravenous fosaprepitant showed similar efficacy to oral aprepitant in adults, a study in children is lacking [54].

*Adverse effects*: Aprepitant and fosaprepitant are commonly well tolerated. The potential for drug interactions is a significant concern for aprepitant/fosaprepitant [55]. A systematic review suggested that the common adverse effects of aprepitant use include hiccups, fatigue, anorexia and constipation; in addition, the risk of serious infections increased from 2% to 6% [56]. The association of the infection rate with the use of aprepitant continues to be unclear and was not shown in prospectively conducted randomized trials [57]. Fosaprepitant is well known to cause injection site reactions, with other adverse effects similar to those of aprepitant, and no difference observed in trials conducted in children [58].

### 4.4. Olanzapine

Olanzapine is a second-generation anti-psychotic that has gained recent wide-spread use as an anti-emetic for CINV prophylaxis and management; its use is also increasing among children.

*Pharmacology*: Olanzapine has a high affinity for the serotonin (5-HT2A/2C, 5-HT3, and 5-HT6), dopamine (D1, D2, D3, and D4), histamine H1 and adrenergic α1 receptors. Its anti-emetic properties are due to its potent antagonism at the serotonergic/dopaminergic/histaminergic receptors (Figure 1). Its anti-nausea activity is likely mediated by similar mechanisms; however, it is poorly understood [23].

The half-life of olanzapine ranges from 21 to 54 h. Its clearance is increased in smokers. Olanzapine has been commonly used off-label in children with psychotic disorders. A pharmacokinetic study of children with childhood-onset schizophrenia, showed similar pharmacokinetic parameters in children compared to adults, on a mg/kg basis [59]. After oral administration, it predominantly binds to albumin and is mainly metabolized by CYPA12, and around 60% of it is renally excreted. Olanzapine has oral bioavailability of 40% and reaches its peak concentration in 6 h.

Olanzapine has comparable pharmacokinetic properties in the fed and fasting states and can be taken with or without food [60]. There was no statistically significant differences between standard oral tablets and disintegrating tablets taken orally or sublingually in the pharmacokinetic properties [61].

*Pediatric dosing and formulations*: The recommended dose of olanzapine for the control of CINV is 0.1–0.14 mg/kg, rounded off to the nearest 1.25 mg. Retrospective real-world data showed that the control of CINV is not dose-dependent [27]. There is a direct linear relationship between olanzapine dose and plasma concentration. When administered at a dose of 12 mg/day, it was shown to approximately occupy 65% of the striatal receptors, and a dose higher than 20 mg/day induced no increase in the clinical response. Clinical responses are maximized at doses between 10 and 15 mg/day in adults. In adults, 5 mg of olanzapine is equally effective as 10 mg of olanzapine against MEC and HEC, with significant decrease in sedation, although nausea control is better with 10 mg [28]. The mean dose of olanzapine administered in pediatric studies was 0.07–0.14 mg/kg. Possibly, the use of a lower dose of olanzapine (0.09 mg/kg) may allow for a reduction in adverse effects without compromising the anti-emetic efficacy [27,62]. Olanzapine is available as 2.5, 5 and 10 mg tablets. Naik et. showed that dose rounding to the nearest 2.5 mg is feasible, safe and effective [63].

*Adverse effects*: Weight gain is the commonly known adverse effect of olanzapine when administered for more than a month at a median dose of 10 mg/day among adults. However, weight gain may not be a significant concern with a short-term use, even though weight gain is more common among children than among adults. Cachexia or a palliative setting might offset a clinically significant weight gain if olanzapine is administered [64,65]. In a meta-analysis of 47 studies including 387 children, evaluating the safety of olanzapine, 78% of the patients showed weight gain, and 48% reported somnolence. Extrapyramidal symptoms were seen in 9% of the patients, while 7% of them reported transaminases elevation, and 4% reported hyperglycemia. No deaths were attributed to olanzapine [66].

### 4.5. Other Anti-Emetics for the Management of CINV in Children

Benzodiazepine, especially lorazepam, is recommended for adults for the primary as well as secondary prophylaxis of anticipatory CINV. In a randomized trial involving adults, the use of lorazepam was associated with a significantly reduced incidence of anticipatory vomiting [67]. On the contrary, a trial evaluating the addition of lorazepam to granisetron in children receiving chemotherapy failed to show any improvement in CINV [68]. Currently, even though lorazepam may be considered for the prophylaxis of anticipatory CINV, despite low-quality evidence, benzodiazepines are not recommended for the management of anticipatory CINV in children [30].

Medical cannabinoids, including tetrahydrocannabinol (THC), dronabinol and nabilone, have been used in adults for refractory CINV; yet, their use is limited by their psychotropic effects. In children, studies evaluating the effects of THC/dronabinol/nabilone were conducted more than three decades ago, when newer anti-emetics like NK1 antagonists and olanzapine were not used. Two prospective cross-over trials in children compared the effects of 10–15 mg/m^2^ of THC with those of prochlorperazine or metoclopramide and demonstrated improved CINV control, while another randomized cross-over trial using 0.5–1 mg of nabilone demonstrated reduced vomiting compared to the use of prochlorperazine [69,70]. The common adverse effects of nabilone include dizziness and drowsiness. A multi-centric retrospective review of the use of nabilone against pediatric CINV, showed CIV control of 50.6% and 53.8% for HEC and MEC, respectively, although adverse effects were observed in 34% of the cases [71]. A similar single-center retrospective review of the use of dronabinol for the management of CINV in children showed that it has been commonly prescribed at 2.5 mg/m^2^/day instead of the recommended dose of 5 mg/m^2^/day and showed moderate CINV control [72]. However, the above agents are currently not in common practice or recommended if newer agents are available.

Besides benzodiazepines and cannabinoids, traditional medicines like ginger root powder have also been extensively evaluated for the management of CINV in adults and children. A randomized trial in children demonstrated that the addition of ginger root powder (20–40 kg: 1000 mg/day; >40 kg: 2000 mg/day) to dexamethasone and ondansetron significantly reduced the incidence and severity of CINV, with no appreciable adverse effects [73]. Besides ginger, other herbs like ginseng, mint oil, etc. have been evaluated for nausea management in pre-clinical settings, and there continues to be a lack of high-quality clinical studies evaluating such traditional medicines for CINV, especially in children [74].

## 5. Optimizing the Combination of Anti-Emetic Agents for CINV Prophylaxis and Important Considerations

A recent update of the CINV guidelines issued by the Pediatric Oncology Group of Ontario provides a summary of the current evidence for anti-emetic use in the pediatric population. In concordance with the ASCO 2020 recommendations, a triplet anti-emetic regimen composed of an NK1 receptor antagonist, a corticosteroid and a 5HT3 antagonist is preferred for HEC, while a doublet regimen consisting of a 5HT3 antagonist and dexamethasone is recommended for MEC [5,75]. Being an anti-emetic with a broad spectrum of action, olanzapine may be a promising addition to the base anti-emetic regimen for HEC [63]. Concerns about possible metabolic and cardiac adverse effects resulted in olanzapine being recommended only conditionally in children receiving HEC. The additive benefit of an NK1 receptor antagonist in anti-emetic regimens containing 5HT3RAs, dexamethasone and olanzapine needs to be explored for HEC.

While data informing guidelines are comprehensive in the coverage of acute CINV, the management of delayed CINV in children is less well understood. Conventionally, combining dexamethasone with 5-HT3 antagonists is recommended against emesis in the delayed phase. Aprepitant, fosaprepitant and olanzapine have all been shown to improve the complete response against delayed CIV when added to 5-HT3RAs/dexamethasone to treat children [21,52,63]. However, aprepitant and fosaprepitant may not be of additive benefit in the control of delayed nausea [22,76]. Among the 5HT3RAs, palonosetron is superior to ondansetron and granisetron in terms of the control of delayed CINV [29]. Thus, if NK1-receptor antagonists and olanzapine cannot be incorporated into the base anti-emetic regimen, palonosetron may be the agent of choice to facilitate the delayed CINV control with MEC and HEC in view of the longer half-life of the drug.

## 6. Anti-Emetics for Multi-Day Regimens

Multi-day chemotherapy regimens used in children lead to an overlap in the acute and delayed phases of CINV, thus blurring the distinction between acute and delayed CINV. It was seen that the maximum incidence of CINV with multi-day cisplatin-based regimens occurs from day 3 to day 5, likely on the account of a sub-optimal anti-emetic administration during this period [77]. Conventionally, daily dexamethasone administration during chemotherapy followed by administration for two days after chemotherapy completion has been used in the prevention of CINV. However, owing to the known adverse effects of prolonged steroid use, dexamethasone-free regimens have been explored in the recent years. The continuation of dexamethasone beyond day 1 may be restricted to those patients receiving HEC regimens with granisetron or ondansetron. For pediatric patients receiving MEC, there is a lack of data regarding the continuation of dexamethasone beyond day 1, although adult trials did not show a consistent benefit [5]. The proven efficacy of olanzapine in delayed CINV control makes it a viable addition to the existing regimen in the case of inadequate control.

## 7. Anti-Emetic Use in the Presence of Hematological Malignancies and Hematopoietic Stem Cell Transplant

CINV and its management in patients with hematolymphoid cancers are entirely different from those in patients with solid organ cancers due to dynamic changes in cytopenia, an increased functional immune-compromised status, increased antibiotic use and a rapid decrease in performance status.

Induction chemotherapy for acute myeloid leukemia causes a typical scenario wherein there is profound neutropenia with a significant risk of sepsis when chemotherapy continues for up to 7 days with 3+7 regimen and, sometimes, for even 10 days with a combination of cytosine arabinoside, daunomycin and etoposide (ADE) [78]. Likewise, in the setting of autologous and allogenic transplants, the regimens last commonly for over 5 days, and again, there is a significant risk of profound and prolonged neutropenia, which is far higher than in the setting of solid tumors [79]. The use of dexamethasone along with chemotherapy may worsen the degree and duration of neutropenia post treatment [80]. Hence, the use of steroids as anti-emetic agents is often discouraged during an active infection or for induction treatment of acute myeloid leukemia. However, the traditional use of 5HT3RAs with/without NK1RAs, is commonly insufficient to control delayed CINV in such scenarios [57]. Hence, the use of alternate agents like olanzapine or the prolonged use of fosaprepitant needs to be explored in these scenarios.

Additionally, corticosteroids are commonly used as part of treatment regimens for acute lymphoblastic leukemia and non-Hodgkin lymphoma. The concomitant use of dexamethasone as an anti-emetic in such scenarios may cause cumulative steroid-related toxicities and needs to be avoided [81].

## 8. Anti-Emetics for Breakthrough Vomiting

By principle, the management of breakthrough CINV needs the addition of a drug that is not incorporated into the base anti-emetic regimen. However, escalating the doses of anti-emetics to those used at high levels of emetogenicity also represents a viable strategy [5]. Although dexamethasone was evaluated in children for breakthrough CINV, its use is based on extrapolation of information on its utility in controlling acute and delayed CINV. Olanzapine was shown to be more efficacious than metoclopramide for breakthrough CINV in children in a phase III RCT [82]. The optimal management of breakthrough vomiting in the setting of a four-drug preventive regimen is unknown and may require adjunctive drugs such as benzodiazepines and phenothiazines [83,84].

## 9. Anti-Emetic Drug Usage Patterns and Guideline Adherence in Children with Cancer

Poor anti-emetic guideline adherence among physicians was found to be associated with poor CINV control [19,20]. Low guideline adherence is more common in the management of pediatric CINV as compared to that of adult CINV. The most frequent causes of lack of adherence include the underuse of dexamethasone [19]. Younger age, treatment in a pediatric rather than an adult oncology clinic, receipt of HEC/MEC and a hematologic malignancy are patient factors noted to be associated with a lack of anti-emetic guideline concordance. The social contributors to non-adherence include lack of guideline awareness and administrative support for guideline implementation [85]. The formulation and implementation of an institutional anti-emetic policy that is concordant with national or international anti-emetic guidelines may promote a better management of CINV [45]. Often, the anti-emetic use is adapted based on the local unavailability of anti-emetic formulations (e.g., the use of a capsule formulation of aprepitant in the absence of suspension formulations) or on individualized choices of patients or physicians.

## 10. Drug Interaction Concerns Regarding Anti-Emetics during Anti-Neoplastic Therapy

The concomitant use of multiple anti-emetic agents, even though efficacious in controlling CINV, is concerning due to drug interactions among anti-emetics themselves and with other chemotherapy and supportive care medications. Such interactions are often clinically significant and need to be kept in mind during clinical use (Table 2) [55,81,86]. Additionally, multiple herbal products exert a significant inhibitory activity on the CYP2D6 enzyme, and their concomitant use may promote drug interactions in children with cancer [87]. Hence, a careful evaluation of the concomitant use of medications including herbal products and alternative medications should be conducted before prescribing anti-emetics.

The use of olanzapine with metoclopramide is to be avoided in view of the enhanced risk of neuroleptic malignant syndrome [88]. Additionally, QTc prolongation is a common adverse effect of multiple classes of anti-emetics like 5HT3RAs, olanzapine and metoclopramide. Hence, QTc prolongation becomes accentuated when these classes of anti-emetics are used together or with other agents like arsenic trioxide or certain tyrosine kinase inhibitors [81]. Similarly, there is a significant potential of interaction with anti-psychotics and anti-depressants due to their overlapping toxicity profiles, although palonosetron and granisetron appear to be safe [86].

Likewise, aprepitant is known to affect the metabolism of multiple drugs due to its inhibitory activity on the CYP3A4 enzyme and affects the bioavailability of multiple chemotherapeutic agents and other supportive care drugs such as cyclosporine, voriconazole, warfarin and oral contraceptive agents [55]. Cyclosporine is commonly used for the prophylaxis of the graft-versus-host disease after allogeneic hematopoietic stem cell transplant (HSCT), while voriconazole and other azole antifungals are commonly used during induction therapy for AML and after HSCT. Hence, drug interactions with anti-emetics play a major role and should be kept in mind during HSCT and AML induction chemotherapy. There are no specific studies that evaluated drug interactions based on the age of the patients, although, when used along with aprepitant, the clearance of dexamethasone is affected to a lesser degree in children compared to adults [18].

## 11. Conclusions and Outlook

Differences in drug metabolism and differences in chemotherapy regimens between adults and children account for the variability in the response to anti-emetic agents between children and adults. Hence, this situation calls for pediatric-specific anti-emetic guidelines. Additionally, while vomiting is an objective and measurable phenomenon, nausea assessment in children is challenging, and validated pediatric nausea assessment tools in various socio-cultural settings should be developed and used for improving the nausea assessment among children [89,90]. The objective symptom assessment of cancer and/or supportive care need to be optimized across countries as per their local socio-cultural aspects, and professional guidelines should also incorporate various adaptations in the use of anti-emetics in the local context [91].

Additionally, the cost-effectiveness of newer anti-emetics is an important aspect that needs to be incorporated while designing institute- or country-specific anti-emetic guidelines. Aprepitant as a third prophylactic agent and olanzapine as a fourth prophylactic agent for CINV were found to be cost-effective from both a high-income and a low-middle income perspective; yet, such studies should be prospectively conducted with newer agents before including the use of these drugs in guidelines [92,93].

It is important to note that even with the available armamentarium of anti-emetics, between 20–30% of children continue to have inadequate CINV control. Traditional medicines like ginger root powder have resulted in a very good control of nausea and vomiting among children receiving HEC [73]. Hence, newer agents for anti-emesis and the efficacy of traditional medicines need to be further explored scientifically with proper randomized trials. Additionally, the role of genetic variants predisposing to CINV and their incorporation into adapted anti-emetic guidelines need to be further studied and optimized.

## Figures and Tables

**Table 1:** Dosage strategies and available formulations of different classes of anti-emetics for children against chemotherapy-induced nausea and vomiting.

S. No.	Class of Anti-Emetics	Individual Agent	Dosage for Children	Available Formulations	Incompatibilities for Intravenous Formulations	Remarks
**1.**	**Steroids**	**Dexamethasone**	6–27 mg/m^2^/day (oral/intravenous) Common dosing strategy: 6 mg/m^2^/dose q6h (in the absence of aprepitant) [17]	Tablet (0.5 mg, 1 mg, 1.5 mg, 2 mg, 4 mg, 6 mg) Oral solution (0.5 mg/5 mL) Oral solution concentrate (1 mg/1 mL) Intravenous formulation (4 mg/mL; 8 mg/mL)	Intravenous dexamethasone is not compatible with doxorubicin, daunorubicin and vancomycin The maximum doses of dexamethasone and ondansetron in solution are 8 mg and 16 mg, respectively, in 50 mL, and 20 mg and 16 mg, respectively, in 100 mL [16]	Dose of dexamethasone is to be reduced by 30% when used with fosaprepitant [18] Oral solution and oral concentrate solution are not available in many countries (commonly, tablet is dissolved in water and administered to younger children) **Contraindications [19,20]:** Induction therapy for acute myeloid leukemia or hematological malignancies treated with steroids (relative contraindications) Patients receiving cranial irradiation; Steroid-related adverse effects;
**2.**	**Neurokinin-1 receptor antagonists**	**Aprepitant**	*Age ≥12 years or weight ≥30 kg* = 125 mg on day1, 80 mg on day 2 and day3 *6 month–12 years* = 3 mg/kg on day 1, 2 mg/kg on day 2 and day 3 [21] *Alternative dosing:* 15–40 kg = 80 mg capsules from day 1 to day 3 [22]	Capsules (125 mg, 80 mg) Powder for oral suspension (available as 125 mg suspended in 4.6 mL of water = 25 mg/mL or extemporaneously prepared) [21]	--	Aprepitant oral suspension formulation is not available in many countries, where for younger children, fosaprepitant or alternative formulations of aprepitant may be used. **Contraindications:** Use with medications with significant interactions (pimozide or cisapride; absolute contraindications) or chemotherapies like ifosfamide (relative contraindications) [23].
		**Fosaprepitant**	*Single Dose* ≥12 years: 150 mg (single dose) 2–12 years: 4 mg/kg (single dose) 6 m–2 years: 5 mg/kg (single dose) *Multi-day: 3 day IV/oral/oral regimen* ≥12 years: 115 mg IV on day 1 followed by oral aprepitant 80 mg on days 2, 3 2–12 years: 3 mg/kg IV on day 1 followed by 2 mg/kg oral aprepitant on days 2, 3 [24]	Intravenous formulation: 150 mg	Intravenous fosaprepitant is not compatible with palonosetron, tropisetron and solutions with magnesium sulphate or calcium gluconate [15]	Dose of fosaprepitant has been variable in trials, between 3 and 4 mg/kg [25,26]. For children receiving multi-day chemotherapy, additional dose of oral aprepitant on day 2 and day 3 is also an alternative dosing strategy recommended by manufacturers [24]. However, clinical trials commonly used single-day dosing with no head-to-head comparison between single-day and multi-day dosing. **Contraindications:** Same as those for aprepitant.
**3.**	**Atypical antipsychotics**	**Olanzapine**	0.1–0.14 mg/kg/day (rounded off to the nearest 1.25/2.5 mg) Maximum: 10 mg [27,28]	Tablet: 2.5 mg, 5 mg, 10 mg (orally disintegrating tablets available) Oral solution (2.5 mg/5 mL, 5 mg/5 mL)		Interactions of olanzapine with other anti-emetics (especially QTc prolongation) should be considered and monitored [29]. Oral solution is commonly not available in many countries. **Contraindications:** Concomitant use of benzodiazepines; Previous grade IV somnolence, QTc > 500 ms or acute pancreatitis [30];
**4.**	**5-Hydroxytryptamine-3 receptor antagonists**	**Ondansetron**	*Intravenous/Intramuscular:* 0.15 mg/kg/dose (maximum 0.45 mg/kg/day i.e., 3 doses per day). Single daily dose of 0.45 mg/kg/day (maximum 32 mg/day) is also acceptable. Alternate dosing: 5 mg/m^2^/day q12h [31] *Oral:* 0.15–0.3 mg/kg/dose (maximum 16 mg/dose, with maximum 0.45 mg/kg/day or 32 mg/day); 0.15 mg/kg is preferable when used in conjunction with other agents for HEC/MEC; otherwise, 0.3 mg/kg/day is preferable if used alone in the presence of low emetogenicity or MEC without steroids Alternate dosing strategy: age 4–11 years (BSA ≤ 0.8 m^2^): 4 mg; age ≥12 years (BSA > 0.8 m^2^): 8 mg	Tablet: 4 mg, 8 mg (orally disintegrating tablets available) Syrup: (2 mg/5 mL, 4 mg/5 mL) Intravenous formulation: 4 mg/2 mL, 2 mg/2 mL (also used intramuscularly)	Intravenous ondansetron is unstable with biologicals like rituximab or gemtuzumab ozogamicin. The maximum doses of dexamethasone and ondansetron in solution are 8 mg and 16 mg, respectively, in 50 mL and 20 mg and 16 mg, respectively, in 100 mL	Even though ondansetron has half-life of 3.5–5.5 h, single daily dose is as efficacious as multiple daily dose for moderately/minimally emetogenic chemotherapy [31,32]. **Contraindications [14]:** Patients with phenylketonuria (due to the presence of aspartame in orally disintegrating tablets); Patients receiving medications like apomorphine; Patients with hypokalemia, hypomagnesemia or heart failure (relative).
		**Granisetron**	*Intravenous:* 40 μg/dose (single dose daily) *Oral:* 20–40 μg/dose (every 12 h) [33,34]	Tablet: 1 mg, 2 mg Oral suspension (extemporaneously prepared): 0.05 mg/mL, 0.1 mg/mL, 0.2 mg/mL Intravenous formulation: 1 mg/mL		Granisetron subcutaneous injectable formulation or transdermal patch is approved for adults but not for children. [32] **Contraindications:** Same as for ondansetron and relative contraindication with concomitant use of medications prolonging QTc
		**Tropisetron**	*Intravenous:* 8–12 mg/m^2^/day or 0.2 mg/kg/day (single daily dose) Oral: 0.2 mg/kg/day [35]	Tablets: 5 mg. 2 mg Intravenous formulation: 1 mg/mL	Intravenous formulation is incompatible with fosaprepitant [15]	Tropisetron is not available in many countries, and oral suspension formulation is commonly made extemporaneously **Contraindications:** Same as for ondansetron, and relative contraindication with concomitant use of medications prolonging QTc
		**Palonosetron**	*Intravenous:* <17 years: 20 μg/kg (single dose, maximum: 1.5 mg) ≥17 years: 0.25 mg (single dose) [36]	Intravenous formulation: 0.25 mg/5 mL, 0.25 mg/2 mL	Intravenous formulation is incompatible with fosaprepitant [15]	Oral formulation (capsule) of palonosetron is available in certain countries, although concerns of poor oral bioavailability limit its use [37]. **Contraindications:** Same as for ondansetron, and relative contraindication with concomitant use of medications prolonging QTc

**Table 2:** Clinically significant drug interactions among anti-emetics and between anti-emetics and chemotherapeutic drugs, tyrosine kinase inhibitors or other supportive drugs.

Drug 1	Interacting Drug	Type of Interaction
**A. Anti-emetic–anti-emetic interaction**		
Olanzapine	Metoclopramide	Increased risk of neuroleptic malignant syndrome and extrapyramidal syndrome [88] Combination should be avoided
Dexamethasone	Aprepitant or Fosaprepitant	Increased systemic exposure to dexamethasone due to reduced clearance (due to action on CYP3A4 enzyme) Reduce dosing of dexamethasone by 30% (IV) among children [18]
Olanzapine	Ondansetron/Granisetron	Increased QTc prolongation Avoid use if alternatives available or monitor ECG [81]
**B. Anti-emetic with chemotherapeutic agents or tyrosine kinase inhibitors**		
Aprepitant/Fosaprepitant	Etoposide Cyclophosphamide Irinotecan	Known to increase systemic exposure of these anti-neoplastic agents (due to action on CYP3A4 enzyme) Clinical significance unclear (monitor toxicities) [23]
Aprepitant/Fosaprepitant	Ifosfamide	Increased risk of ifosfamide-induced neurotoxicity Association unclear. Preferably to avoid use in children who developed neurotoxicity [23]
Ondansetron/Granisetron	Arsenic trioxide Capecitabine Lenvatinib Sorafenib Sunitib Dasatinib	Increased risk of QTc prolongation Avoid use with arsenic trioxide Monitor ECG (if used with the other drugs) [81]
Olanzapine	Arsenic trioxide	Increased risk of QTc prolongation Avoid use [81]
Granisetron	Vincristine	Increased risk of constipation Clinical significance unclear
Dexamethasone	Imatinib Dasatinib Sunitinib Sorafenib	Decreased systemic exposure to tyrosine kinase inhibitors (due to upregulation of CYP3A4) [81] Consider up-titration of dose if long-term concomitant use anticipated (e.g., increase dose of imatinib by 50%)
**C. Anti-emetic and supportive care medications**		
Aprepitant/Fosarepitant	Cyclosporine	Increased systemic exposure to cyclosporine Titrate dose of cyclosporine according to serum levels and monitor toxicities [23,81]
Aprepitant/Fosaprepitant	Voriconazole	Increased systemic exposure to aprepitant [23] Clinical significance unclear
Aprepitant/Fosaprepitant	Warfarin Oral contraceptives	Increased systemic exposure to warfarin and oral contraceptives [81] Use of alternative methods of contraception monitor INR with warfarin and watch for bleeding manifestations

## Data Availability

No new data were created or analyzed in this study. Data sharing is not applicable to this article.
